# The effect of dietary supplementation of *Lycium barbarum* leaves on the growth performance, organ indexes and intestinal microflora of rats

**DOI:** 10.3389/fvets.2024.1416793

**Published:** 2024-07-31

**Authors:** Yindi Guo, Jie Liu, Qiang Tuo, Dongtao Zhang, Metha Wanapat, Guosheng Xin

**Affiliations:** ^1^School of Life Science, Ningxia University, Yinchuan, China; ^2^Ningxia Feed Engineering Technology Research Center, Ningxia University, Yinchuan, China; ^3^Key Lab of Ministry of Education for Protection and Utilization of Special Biological Resources in Western China, Ningxia University, Yinchuan, China; ^4^Agricultural College, Ningxia University, Yinchuan, China; ^5^Khon Kaen Univ, Fac Agr, Trop Feed Resources Res & Dev Ctr TROFREC, Dept Anim Sci, Khon Kaen, Thailand

**Keywords:** *Lycium barbarum* leaves, *Lycium barbarum* polysaccharides, microbiota, functional feed, organ weight

## Abstract

This study was conducted to investigate both fruit and different levels of leaf supplementation on the growth performance, organ indices and intestinal microflora of rats. Twenty-five healthy male Sprague–Dawley rats were randomly divided into five groups. The rats in the control (NC) and positive control (PC) groups were fed by gavage a basal diet and a basal diet with 4 g/kg of *L. barbarum* fruit homogenate, respectively. The test (LD, MD, and HD) groups were fed basal diets with additional 2, 4, and 8 g/kg of *L. barbarum* leaf homogenate, respectively. The feeding period was 35 d. The result revealed that the rats in the LD group had the highest average weight gain (*p* < 0.05). The cardiac and renal indexes in the LD and MD groups were significantly higher than in NC group, respectively (*p* < 0.05). Diversity analysis revealed that adding low concentrations of *L. barbarum* leaf homogenates markedly reduced the Shannon index of the rats cecum (*p* < 0.05). The relative abundance of Verrucomicrobiota was higher in the LD group than those in other groups (*p* < 0.05). The relative abundance of Actinobacteriota was found significantly higher in PC group than others (*p* < 0.05). The relative abundance of *Akkermansia* in LD group was the highest (*p* < 0.05). The relative abundance of Romboutsia in the PC group was considerably higher than that in other groups. The relative abundance of Candidatus_Saccharimonas in the supplementation groups was appreciably lower than those found in other groups. The relative abundance of Alloprevotella was significantly lower in PC, LD, and MD groups than in NC and HD groups (*p* < 0.05). The relative abundance of Oscillibacter was significantly higher in HD group than in other groups (*p* < 0.05). Thus, *L. barbarum* leaf homogenate fed to rats could increase their growth performance, internal organ weights and additionally enhance the relative abundance of beneficial bacteria. Therefore, based on the obtained data in the current study, a dose of *L. barbarum* leaf homogenate supplemented with 2 g/kg in diet is recommended, however, further studies are required to confirm, especially in animals.

## 1 Introduction

*Lycium barbarum*, a perennial shrub with unique drought-resistant characteristics, is widely distributed in northwestern China. Studies have shown that *L. barbarum* is rich in several active ingredients, such as *L. barbarum* polysaccharides (LBP), betaine, polyphenols, amino acids, and trace elements (1), which are crucial for boosting immunity, relieving inflammation, and protecting the liver (2). Furthermore, *L. barbarum* is widely used in health products, functional foods, and feed additives (3). Compared to its fruit, the leaves have been neglected as a byproduct, with lower utilization levels for a long time (4). However, the chemical composition of *L. barbarum* leaves in terms of their active ingredients, nutrients, and trace elements is similar to that of its fruits and has the same utility value (4). Recently, *L. barbarum* leaves are preferred by increasing number of consumers as vegetable, tea, and food ingredients. LBP, the main active ingredient in *L. barbarum* leaves, has been shown to improve immune responses, maintain the structural integrity of the intestinal tract, increase the relative abundance of beneficial bacteria in the intestinal tract microflora, and enhance animal growth performance (5). Yin et al. (6) showed that adding LBP to the diet improved the structure of intestinal microflora and improved the growth performance and immunity of weaned piglets. Mo et al. (7) showed that adding 2 g/kg LBP to the diet significantly improved the growth performance of grass carp and Nile tilapia. Liang et al. (8) found that supplementing the diet of mice with 200 mg/kg LBP increased the relative abundance of beneficial intestinal bacteria and altered the structure of intestinal microflora. The leaves of *L. barbarum* have high application potential in livestock and poultry production. Relatively few studies focuse on the supplementation of *L. barbarum* leaves in diets, with the majority of research concentrating on LBP functional aspects. The use of *L. barbarum* leaves in animals and their subsequent utilization as a feed source is devoid of theoretical direction, which in turn restricts the industrial application of *L. barbarum leaves* in production.

This study investigated the effects of different doses of *L. barbarum* leaf homogenates on the body weight, visceral organ index, and intestinal microorganisms, and further evaluated its effects on rat growth performance, organ development, and intestinal health, with an aim to provide a theoretical basis and technical support for developing and utilizing *L. barbarum* leaves as a feed resource.

## 2 Materials and methods

### 2.1 Test material

Male Sprague–Dawley (SD) rats were purchased from Shaanxi Nuoyou Biotechnology Co. (Xian China). The rats were fed a commercial diet as their basal feed, which was composed of imported fish meal, imported chicken meal, soybean meal, corn, meat and bone meal etc., with the following nutritional values listed on its label: dry matter 90%, crude protein 18%, crude fat 4%, crude fiber 5%, crude ash 8%, calcium 1.0–1.8%, phosphorus 0.6–1.2%. *L. barbarum* leaves and its fruits were collected in Zhongning County, Zhongwei City, Autonomous Ningxia Hui Region. A homogenate was made by dissolving 720 g of *L. barbarum* leaves or fruit into 1 L of distilled water, with a concentration of 0.72 g/mL. The freshly harvested *L. barbarum* leaves and *L. barbarum* were homogenized in a grinding machine and stored at 4°C (Table 1).

**Table 1:** Nutrient content of *Lycium barbarum* leaves and fruits (DM basis).

Items	*L. barbarum* leaves	*L. barbarum* fruits
Conventional nutrients		
CP	27.80 (A)	13.79 (A)
EE	1.10 (C)	2.23 (A)
NDF	31.07 (A)	10.29 (A)
ADF	13.33 (A)	10.58 (A)
Ash	17.83 (A)	13.95 (A)
Functional components		
Polysaccharide	9.56 (A)	2.93 (A)
Flavonoid	1.32 (A)	0.02 (A)
Betaine	1.36 (B)	1.11 (B)

A = Hou et al. (58), B = Ma et al. (59), C = Zhang (60).

### 2.2 Experimental design and rat management

Twenty-five healthy male SD rats (5–6 weeks old) were selected and randomly divided into five groups based on similar body weights, and each group had five rats as replicates. The NC and PC groups were administered the basal diet containing 4 g/kg (kg is the weight of the rat) of *L. barbarum* fruit homogenate by gavage and the rats in LD, MD, and HD groups received a basal diet with 2 g/kg, 4 g/kg, and 8 g/kg of *L. barbarum* leaves, respectively, which were homogenized by gavage.

Feeding trials were conducted in the Zhixing Building of Ningxia University, where the test site was naturally lit, dried, and ventilated. Each rat in the NC, PC, LD, MD and HD groups received 1 mL (normal saline), 1 mL, 0.5 mL, 1 mL, 2 mL (the positive control group received *L. barbarum* fruit homogenate, and the experimental group received *L. barbarum* leaves homogenate by gavage). The experiment was conducted over 35 d. The animals were housed for 7 d for acclimatization and 28 d for the experimental period. Rats were provided water *ad libitum*, fed, and managed according to routine procedures.

### 2.3 Sample collection

The rats were weighed on the last day of the entire experiment after fasting for 12 h, anesthetized, and fixed on a dissecting board in a comatose state. Liver, spleen, kidney, thymus, and testis were weighed and washed with pre-cooled saline, and the index of each organ was calculated. The cecum contents were collected in sterile Eppendorf (EP) tubes, rapidly snap-frozen in liquid nitrogen, and then transferred to a − 80°C freezer for later tests.

### 2.4 Measurement indicators and methods

#### 2.4.1 Growth performance measurement

The initial body weight of the rats was measured on day 1 of the test and the final body weight was also measured on day 28. The amount of food consumed and the amount of feed that remained were used to calculate the daily feed intake. Additionally, the average daily weight gain (ADG), average daily feed intake (ADFI), and ADFI/ADG (F/G) were computed.

ADG = (final weight − initial weight)/number of test days.

ADFI = feed consumption during the feeding period/number of test days.

F/G = ADFI/ADG.

#### 2.4.2 Organ index measurement

The live weight of the rats was recorded at the end of the experiment, and the heart, liver, spleen, kidney, thymus, and testes were aseptically removed after the rats were euthanized under anesthesia. Blood was aspirated using filter paper, and the organ index was calculated (9).

#### 2.4.3 Determining the intestinal microorganisms

The bacterial community structure was characterized using 16S rRNA gene sequencing. The rat cecum contents were collected, stored at −80°C in a freezer, and sent to Novozymes (Beijing, China) for 16S microbial bioinformatics analysis. Polymerase chain reaction (PCR) products were electrophoresed, purified, and mixed on a 2% gel. After the PCR products were completely mixed, PCR was performed and the products were recovered as target band reagents. The samples were clustered with 97% consistency in operational taxonomic unit (OTU) clustering, and the sequence with the highest OTU abundance was selected as the representative sequence for alpha diversity index analysis.

### 2.5 Statistical analysis

Raw data were processed using Excel 2016, and SPSS 26.0 was used for one-way analysis of variance and Duncan’s method. The correlation analysis of growth performance, organ index, and gut microbiota was investigated using Spearman’s correlation analysis, and *p* < 0.05 was considered statistically significant.

## 3 Results

### 3.1 Effect of *Lycium barbarum* leaves on the growth performance of rats

The difference in initial weight of rats between the five groups was insignificant (Table 2; *p* > 0.05). The final weight and ADG of rats in the LD group (338.63 g and 6.25 g/d, respectively) were significantly higher than those in the NC, MD, and PC groups (*p* < 0.05). The ratio of ADFI and feed weight was the lowest at 5.24; however, the differences between all groups were insignificant (*p* > 0.05).

**Table 2:** Effects of *Lycium barbarum* leaf on the growth performance of rats.

Items	Groups					SEM	*p*-value
	NC	PC	LD	MD	HD		
IW/g	176.09	177.77	176.14	177.64	176.26	0.32	0.217
FW/g	294.96^b^	307.04^b^	338.63^a^	298.82^b^	313.12^ab^	5.10	0.035
ADG(g/d)	4.57^b^	4.97^b^	6.25^a^	4.66^b^	5.26^ab^	0.20	0.032
ADFI(g/d)	34.31	35.96	32.34	34.54	32.68	0.76	0.662
F/G	7.51	7.60	5.24	7.54	6.22	0.38	0.217

NC, control group; PC, positive control group; LD, MD, HD, test groups; IW, initial weight; FW, final weight; ADG, Average daily weight gain; ADFI, Average daily feed intake; F/G, ADFI/ADG; SEM, standard error. Significant differences are indicated by different lowercase letters in the same row (*p* < 0.05), whereas the same lowercase letters indicate no significant differences (*p* > 0.05).

### 3.2 Effect of *Lycium barbarum* leaves on organ indices of rats

The supplementation of different levels of *L. barbarum* leaves had markedly affected the cardiac and renal indices of the rats (Table 3; *p* < 0.05); however, the differences in the indices of the liver, spleen, thymus, and testicular tissues were insignificant (*p* > 0.05). The cardiac index was significantly higher in the PC and LD groups than that in the NC group (*p* < 0.05) and tended to decrease progressively with an increase in the amount of *L. barbarum* leaves supplementation in the diet. The kidney index of rats treated with 4 g/kg *L. barbarum* leaf homogenate was the highest (*p* < 0.05) and showed an increasing trend in the beginning, with the increase in the added level of *L. barbarum* leaves, but rapidly decreased with additional supplementation. The spleen and thymus indices of the rats in the experimental groups showed an upward trend; however, the differences between the groups were insignificant (*p* > 0.05).

**Table 3:** Effect of *Lycium barbarum* leaves on organ index in rats.

Items	Groups					SEM	*p*-value
	NC	PC	LD	MD	HD		
Cardiac index (%)	0.36^c^	0.4.2^a^	0.41^ab^	0.38^abc^	0.3.6^bc^	0.008	0.033
Liver index (%)	3.14	3.31	2.93	3.23	3.13	0.058	0.331
Spleen index (%)	0.18	0.16	0.18	0.21	0.17	0.005	0.132
Kidney index (%)	0.89^b^	0.94^ab^	0.90^b^	1.00^a^	0.96^ab^	0.013	0.048
Thymus index (%)	0.15	0.16	0.14	0.16	0.17	0.006	0.772
Testis index (%)	1.15	1.05	1.01	1.15	1.03	0.022	0.083

NC, control group; PC, positive control group; LD, MD, HD, test groups; SEM, standard error. Different lowercase letters in the same row indicate a significant difference (*p* < 0.05), whereas the same lowercase letters indicate no significant differences (*p* > 0.05).

### 3.3 Effect of *Lycium barbarum* leaves on the levels of intestinal microorganisms in rats OTU analysis

**Figure 1:**
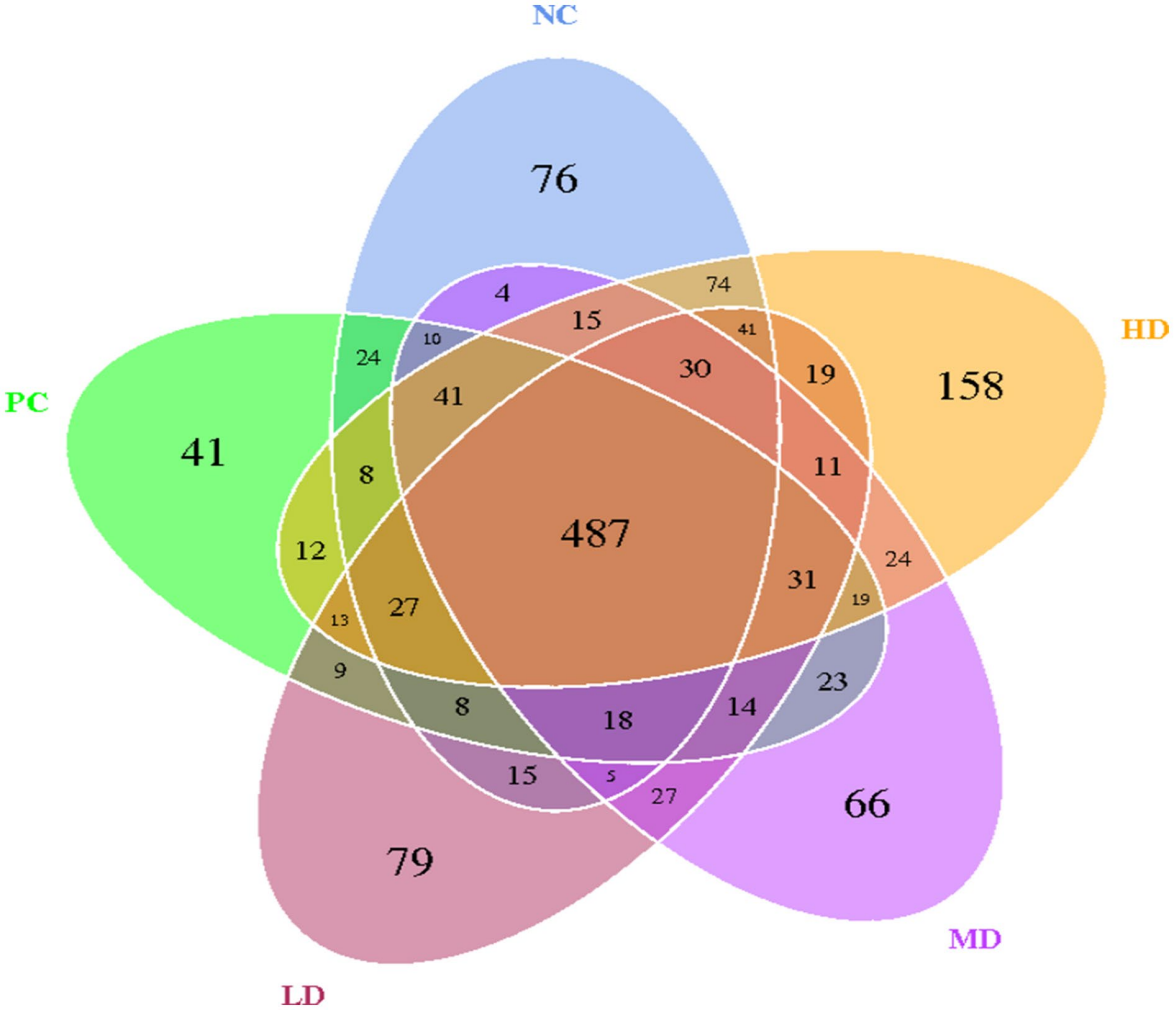
Venn map of the intestinal flora at the OTU level.

The OTU Venn diagram obtained by clustering the samples from the five groups revealed 487 identical OTUs (Figure 1). A total of 76 characteristic OTUs, 41 unique OTUs, 79 exclusive OTUs, 66 specific OTUs, and 158 own OTUs were found in the NC, PC, LD, MD, and HD groups, respectively.

#### 3.3.1 Alpha diversity analysis

**Figure 2:**
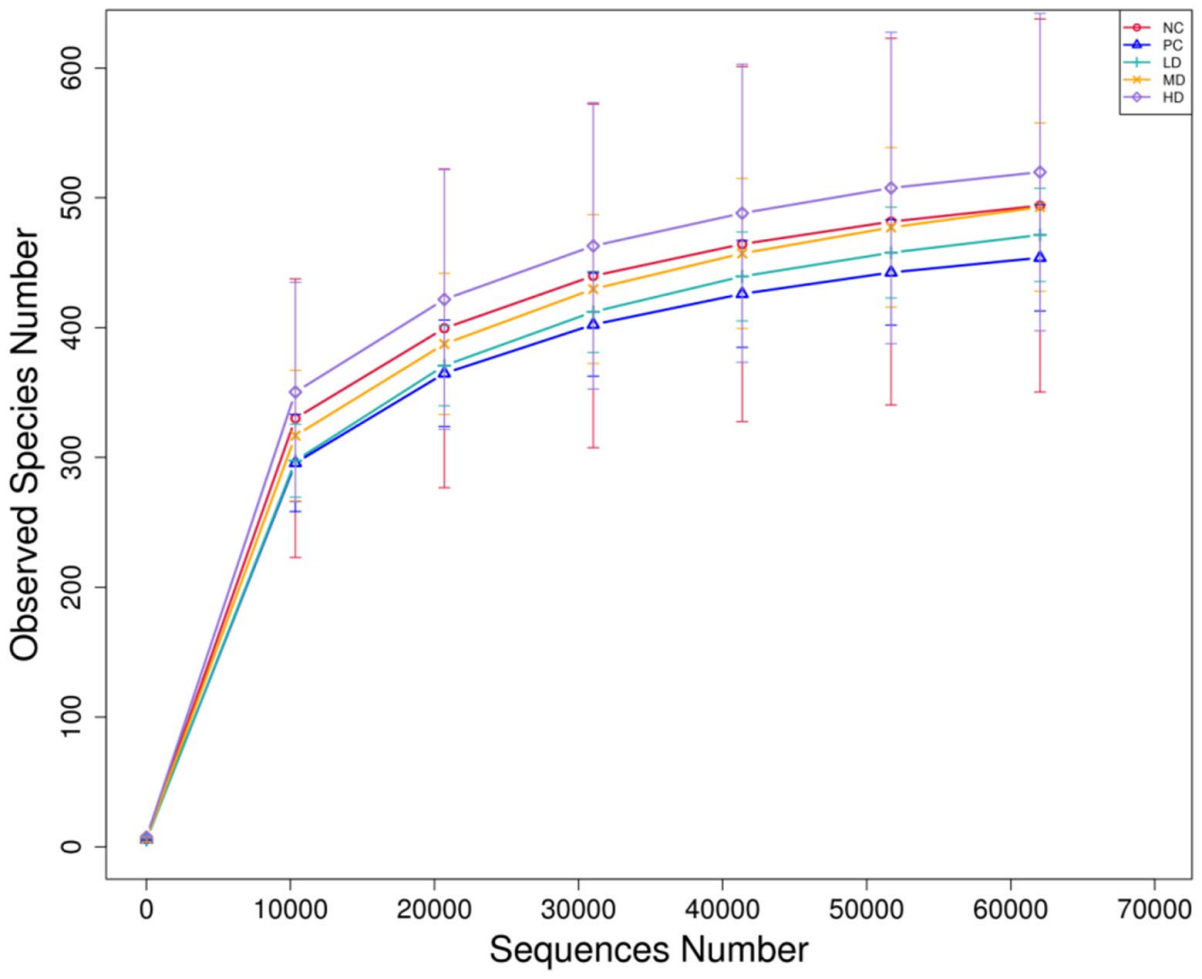
Sparse curve of cecal flora in rats.

Figure 2 shows the results of the sparse curve. As the number of sequencing strips increases, the curve becomes flatter, indicating that the amount of sequencing is gradually becomes reasonable. Table 3 shows that the addition of different amounts of *L. barbarum* leaf homogenates to the basal diet significantly affected the Shannon index of rat gut flora, which was markedly lower in the LD group than in the NC group (*p* < 0.05) and tended to increase with increasing doses of *L. barbarum* leaves homogenates in the test groups (*p* > 0.05; Table 4).

**Table 4:** Effects of *Lycium barbarum* leaves on the analysis of bacterial diversity in rats.

Items	Groups					SEM	*p*-value
	NC	PC	LD	MD	HD		
observed_species	563.50	471.25	486.25	492.80	465.00	13.05	0.123
Shannon	5.15^a^	4.15^abc^	3.22^c^	3.81^bc^	4.94^ab^	0.22	0.015
Simpson	0.90	0.81	0.79	0.78	0.92	0.03	0.298
Chao1	599.98	504.55	533.07	510.61	500.53	14.08	0.129
ACE	605.49	505.73	533.15	514.65	538.99	12.51	0.057
PD-whole-tree	39.62	32.89	52.15	55.37	35.16	3.87	0.224

NC, control group; PC, positive control group; LD, MD, HD, test groups; SEM, standard error. Different lowercase letters in the same row indicate a significant difference (*p* < 0.05), whereas the same lowercase letters indicate no significant differences (*p* > 0.05).

#### 3.3.2 Effect of *Lycium barbarum* leaves on the structure of intestinal microorganisms in rats

**Figure 3:**
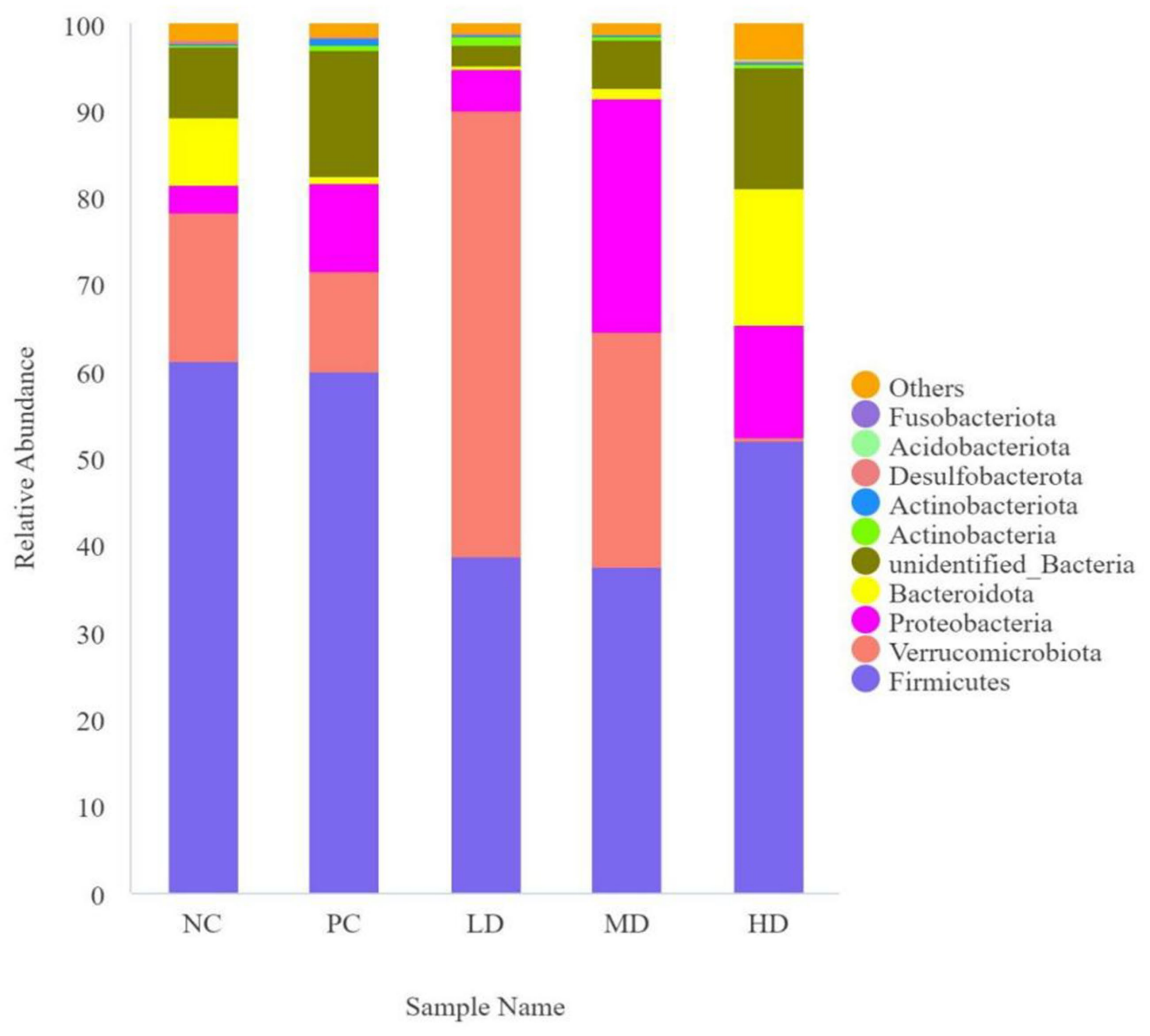
Map of the relative abundance of *Lycium barbarum* leaf compared to the intestinal flora of different phylum in rats.

Figure 3 shows that the phylum level results along with an analysis of the compositional structure of the gut microbiota. The dominant bacteria were Firmicutes and Verrucomicrobiota. Table 5 shows that the relative abundance of Verrucomicrobiota was significantly higher in LD than in NC, PC, and HD (*p* < 0.05), and that the relative abundance of Verrucomicrobiota in the experimental groups rapidly decreased (*p* < 0.05) with increasing doses of *L. barbarum* leaves homogenates. The relative abundance of Actinobacteriota was significantly higher in the PC group than in the other groups (*p* < 0.05). The relative abundance of unidentified bacteria was significantly lower in the LD group than in the PC group (*p* < 0.05) and increased as more *L. barbarum* leaves were added.

**Table 5:** Relative abundance at gate level.

Items	Groups					SEM	*p*-value
	NC	PC	LD	MD	HD		
Firmicutes	61.26	59.94	38.69	37.46	52.04	4.82	0.398
Verrucomicrobiota	17.16^b^	11.54^b^	51.33^a^	27.08^ab^	0.45^b^	5.46	0.018
Proteobacteria	3.16	10.26	4.82	26.90	12.95	3.50	0.326
Bacteroidota	7.62	0.76	0.35	1.13	15.59	2.08	0.068
unidentified_Bacteria	8.29^abc^	14.45^a^	2.51^c^	5.56^bc^	13.95^ab^	1.52	0.022
Actinobacteria	0.13	0.71	0.90	0.49	0.47	0.11	0.251
Desulfobacterota	0.26	0.16	0.08	0.04	0.22	0.03	0.140
Actinobacteriota	0.26^b^	0.69^a^	0.27^b^	0.20^b^	0.23^b^	0.06	0.037
Acidobacteriota	0.00	0.00	0.00	0.00	0.09	0.02	0.452
Fusobacteriota	0.00	0.04	0.01	0.00	0.05	0.01	0.729
Others	1.89^b^	1.44^b^	1.06^b^	1.13^b^	3.97^a^	0.32	0.008

NC, control group; PC, positive control group; LD, MD, HD, test groups; SEM, standard error. Different lowercase letters in the same row indicate a significant difference (*p* < 0.05), whereas the same lowercase letters indicate no significant differences (*p* > 0.05).

**Figure 4:**
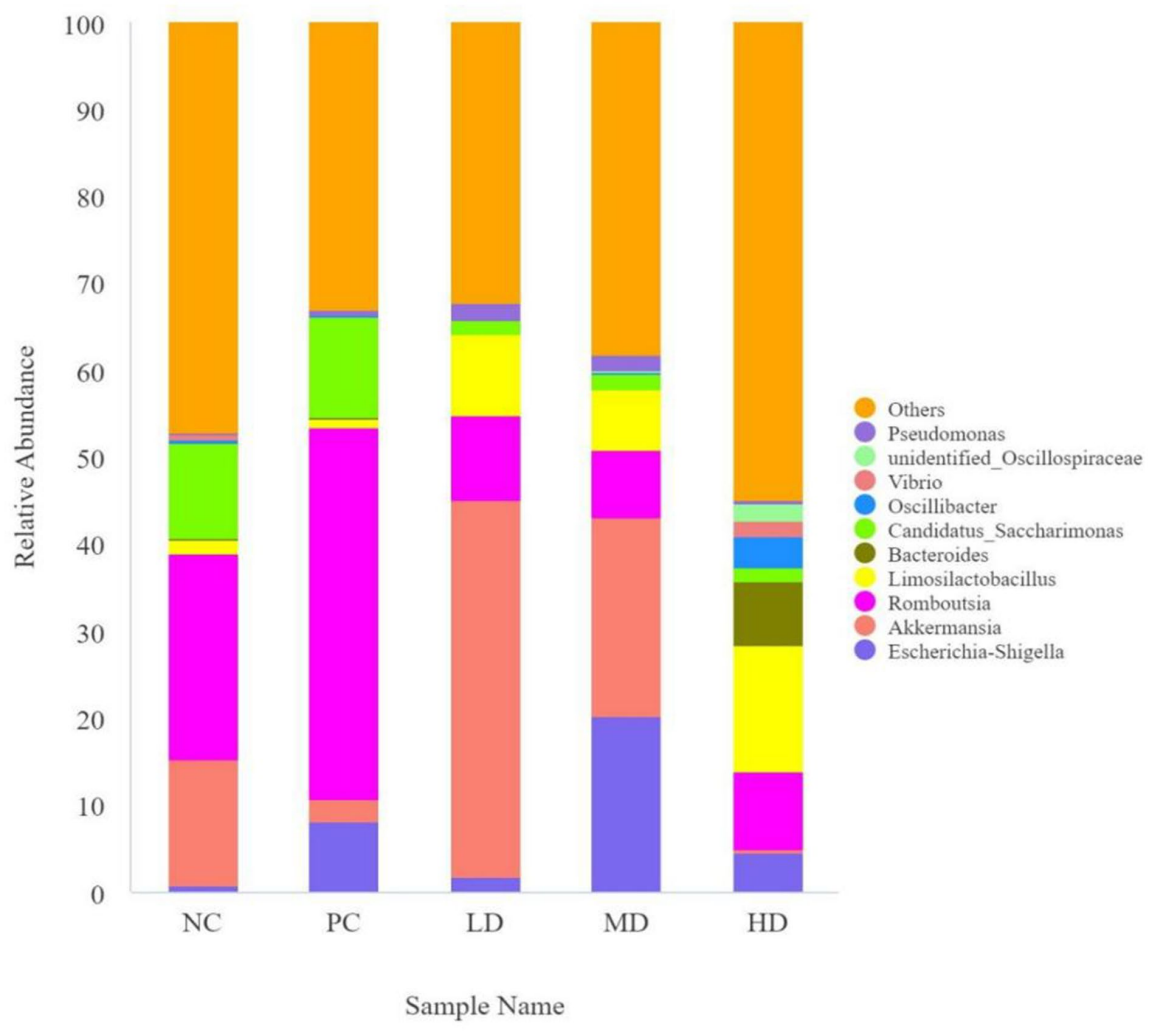
Map of the relative abundance of *Lycium barbarum* leaves compared to the intestinal flora of different genera in rats.

*Akkermansia* and Romboutsia were the dominant genera (Figure 4). Table 6 shows that the LD group had the highest relative abundance of *Akkermansia*, which was significantly higher than that of the PC group (*p* < 0.05). The relative abundance of Romboutsia was higher in the PC group than that in the other groups (*p* < 0.05). The relative abundance of Candidatus_Saccharimonas significantly decreased by the addition of *L. barbarum* leaves (*p* < 0.05). The relative abundance of Alloprevotella in the LD, MD, and PC groups rapidly decreased compared with that in the NC and HD groups (*p* < 0.05). The relative abundance of Oscillibacter was higher in the HD group than that in the other groups (*p* < 0.05).

**Table 6:** Relative abundance of flora of different genera.

Items	Groups					SEM	*p*-value
	NC	PC	LD	MD	HD		
Escherichia-Shigella	0.82	8.12	1.81	20.38	4.50	3.88	0.512
Akkermansia	14.40^ab^	2.67^b^	43.37^a^	22.85^ab^	0.49^b^	5.16	0.043
Romboutsia	23.74^b^	42.77^a^	9.61^b^	7.63^b^	8.90^b^	4.04	0.001
Limosilactobacillus	1.69	0.95	9.40	6.93	14.58	2.58	0.501
Bacteroides	0.15	0.15	0.09	0.08	7.30	1.17	0.248
Candidatus_Saccharimonas	10.88^a^	11.65^a^	1.46^b^	1.74^b^	1.58^b^	1.51	0.013
Alloprevotella	0.62^a^	0.03^b^	0.02^b^	0.00^b^	0.67^a^	0.10	0.025
Oscillibacter	0.32^b^	0.14^b^	0.08^b^	0.24^b^	3.55^a^	0.45	0.055
Anaerostipes	0.36	0.28	0.08	0.04	1.11	0.14	0.126
Phascolarctobacterium	0.19	0.25	0.07	0.72	1.23	0.18	0.282
Others	46.83	33.02	34.02	39.41	56.11	3.91	0.357

NC, control group; PC, positive control group; LD, MD, HD, test groups; SEM, standard error. Different lowercase letters in the same row indicate a significant difference (*p* < 0.05), whereas the same lowercase letters indicate no significant differences (*p* > 0.05).

### 3.4 Correlation of growth performance, organ index, and gut microbiota

**Figure 5:**
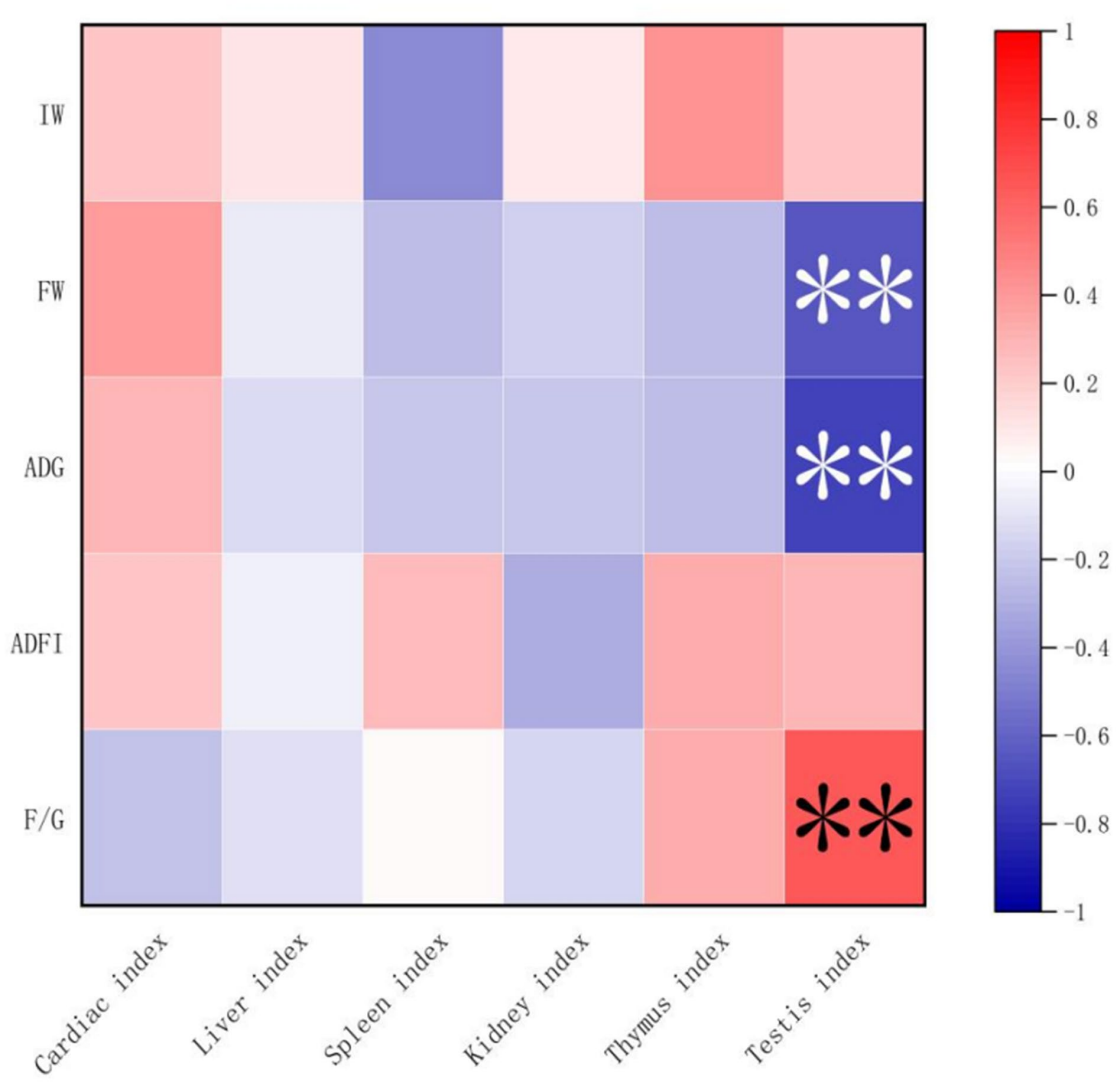
Correlation between growth performance and organ index. IW, initial weight; FW, final weight; ADG, Average daily weight gain; ADFI, Average daily feed intake; F/G, ADFI/ADG. “*” denotes significant correlation, and “**” indicates an extremely significant correlation.

**Figure 6:**
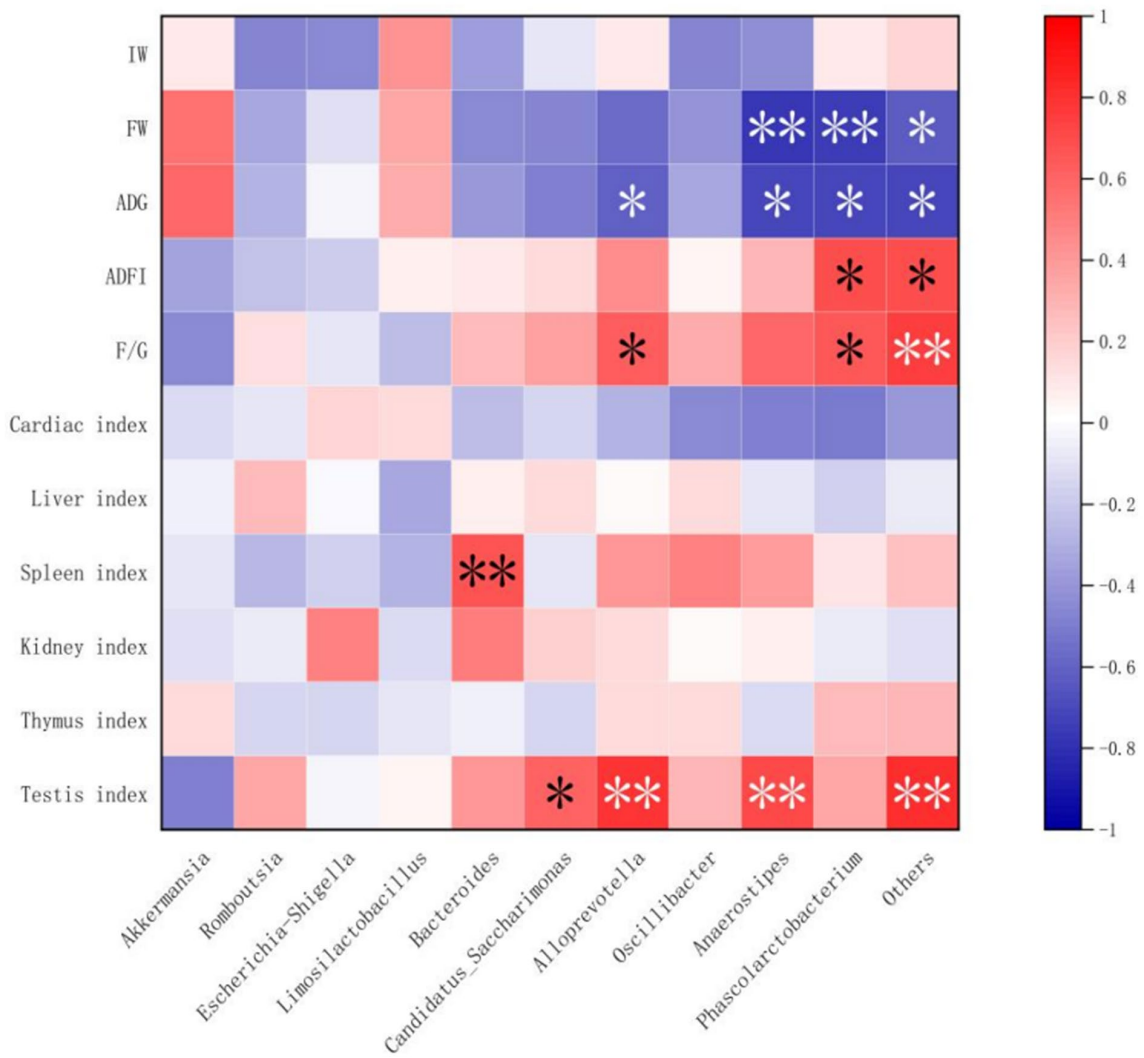
Correlations between growth performance and organ indices and intestinal flora (at genus level). IW, initial weight; FW, final weight; ADG, Average daily weight gain; ADFI, Average daily feed intake; F/G, ADFI/ADG. “*” and “**” denoted extremely significant correlation, respectively.

Spearman’s correlation analysis was performed, and heat maps were generated (Figures 5, 6). A correlation was found between the supplementation quantities of *L. barbarum* leaves and the final weight, daily weight gain, testicular index, spleen index, and intestinal microorganisms at the genus level in rats. Testicular index was negatively correlated with the final weight and ADG (*p* < 0.01) and positively correlated with F/G (*p* < 0.01). Bacteroides showed a highly significant positive correlation with spleen index (*p* < 0.01) and testicular index with Candidatus_Saccharimonas, Alloprevotella, Anaerostipes, and other bacteria (*p* < 0.05). Notably, the F/G was positively correlated with Alloprevotella, Phascolarctobacterium, and other bacteria (*p* < 0.05), and the ADFI was significantly and positively correlated with Phascolarctobacterium and other bacteria (*p* < 0.05). The final weight of the rats was significantly negatively correlated with Anaerostipes, Phascolarctobacterium, and other bacteria (*p* < 0.01), and daily weight gain was significantly negatively correlated with Alloprevotella, Anaerostipes, Phascolarctobacterium, and other bacteria (*p* < 0.05).

## 4 Discussion

Growth performance is an important indicator in animal production, and the average daily gain of animals reflects their growth profile (10). Adding *L. barbarum* leaves to the animal diet significantly increased the body weight gain of rats in this study. LBP and betaine are the primary active ingredients in *L. barbarum* leaves (11). Several studies have suggested that active substances such as LBP and betaine enhance animal growth (12). Long et al. (13) found that LBP added to the diets of broilers could increase the ADG and markedly improve growth performance, and recommended a supplementation level of 2000 mg/kg. Zhang et al. (14) found that adding LBP to the diet of Nile tilapia can appreciably increase growth rate, relative length, and body weight gain, and promote growth performance. Chen et al. (15) confirmed that LBP significantly increased the ADG of weaned piglets. Yang et al. (16) suggested that adding betaine to the diet could improve the growth performance in fattening pigs, and the present study showed similar results in improving growth performance compared to those reported by previous studies. The correlation analysis between growth performance and organ weights of rats in this experiment showed a highly significant and negative relationship between the final body weight, daily weight gain, and testicular index, which may be attributed to higher body weight gain than other reproductive organs in later growth stages. Zhou et al. (17) previously reported similar results. Adding *L. barbarum* leaves to diets has been shown to improve animal growth and increase the weight of immune organs. Zhao et al. (18) reported that dietary supplementation with 0.2% LBP enhances the abundance of beneficial gut bacteria by improving the activity of the intestinal microbiome, thus promoting growth performance of animals. Liu et al. (19) found that LBP promoted broiler growth performance by enhancing immune function. Ma et al. (20) showed that betaine stimulates glucose utilization in animal muscle cells, thereby improving growth performance. The present study examined the variation in the structure of the cecum flora of rats after adding different doses of *L. barbarum* leaf homogenates using the Illumina MiSeq sequencing platform and using 16S rRNA sequencing technology. However, the interaction between intestinal microorganisms and functional substances in LBP is unknown, and further studies are required to understand the specific molecular mechanisms.

The organ index reflects the development and functioning of animals; the higher the relative weight of an animal organ, stronger its function (21). In the present study, adding *L. barbarum* leaves sharply increased cardiac and renal indices in rats probably because betaine in the leaves increased growth factor levels in the blood of rats, promoted heart development, and reduced kidney inflammation. Fiorito et al. (22) reported that betaine protects and improves the functions of internal organs. Yu et al. (23) found that LBP has a protective effect on the kidneys. Xie et al. (24) showed that 400 mg/kg LBP reduced kidney inflammation and renal tissue apoptosis in mice by activating the nuclear factor E2-related factor 2 and oxygenase-1 (HO-1) signaling pathways, thereby improving the immune capacity of rats. Deng et al. (25) found that adding LBP to the diet had a consistent effect on the spleen index in mice, consistent with that of previous studies. This is likely because of LBP’s ability to alleviate the inflammatory response and increase antibody counts that ultimately strengthens humoral immunity. Ruo et al. (26) reported that LBP regulates the development and differentiation of immune cells, such as T lymphocytes, B lymphocytes, macrophages, and dendritic cells and downregulates the inflammatory immune response, and inhibits the secretion of pro-inflammatory cytokines. Additionally, dietary LBP has been shown to increase the activity of antioxidant liver enzymes and their gene expression, reduce liver inflammation, increase antibody production, and improve immune function (27). Ding et al. (28) demonstrated that LBP improves immune organ function by modulating intestinal microbiota structure. In this study, the spleen index was significantly and positively correlated with Bacteroides. The spleen is the largest secondary lymphoid organ and performs various immune functions (29). Stimulation of multiple immune cells or multiple pathways may indirectly activate the NF-κB signaling pathway to promote humoral and cellular immunity (30). Bacteroides primarily produce short-chain fatty acids (SCFAs) that can regulate the immune function of rats by increasing mucoprotein 2 expression (31). Liu et al. (19) found that adding 4 g/kg LBP to the diet markedly increased the immune organ index in broiler chickens, which is inconsistent with the results of this study and may be related to the LBP dosage in *L. barbarum* leaves and the animal species used in the study.

The large number of bacteria in the intestinal tract of animals plays a key role in intestinal and host health and can affect the growth, development, nutrient absorption, and immunity of animals (32). The diversity of intestinal flora is closely related to intestinal homeostasis, and the higher the index of flora diversity, the more difficult it is to destroy balance of flora balance (33). However, the composition of the intestinal microbial community is highly dependent on diet (34). Additionally, Fang et al. (35) reported that adding LBP changed the structure of the intestinal microbiota in mice, increased the abundance of beneficial bacteria, and inhibited the proliferation of harmful bacteria, which were similar to the results of the present study when *L. barbarum* leaves were used at different concentrations; however, the distribution pattern and stability of the intestinal microorganisms were disturbed. Sun et al. (21) added different LBP values to fish diets and found that the Shannon index showed an upward trend with increasing LBP supplementation dose, which is consistent with the results of this study. However, the Shannon index was significantly lower in the LD group, possibly because the LBP content was lower in the low-dose homogenate of *L. barbarum* leaves, which inhibited the growth of some gut microbiota. Sun et al. (21) found that the diversity of intestinal microorganisms (Chao1 and Simpson) in cultured fish slightly decreased with the addition of different levels of LBP to the diet, which is consistent with the results of the present study. In conclusion, infusion of *L. barbarum* leaves in rats slightly reduced the diversity of the gut microbiome.

The predominant bacterial phyla in the rat cecum were Firmicutes and Verrucomicrobiota, which is in agreement with the findings of Ding et al. (36). In the present study, the relative abundance of Verrucomicrobiota in the intestinal tract of rats was significantly increased by adding low-dose homogenate of *L. barbarum* leaves. Bai et al. (37) found that administering 150 mg/kg of *L. barbarum* polysaccharides to leaves significantly increased the relative abundance of Verrucomicrobiota, which is consistent with the results of the present study. Verrucomicrobiota is a potentially beneficial bacterium that can utilize gastrointestinal mucin as an energy source and protect the gut from pathogens by increasing the number of mucin-producing goblet cells in rats (38, 39). The relative abundance of Actinobacteriota was markedly increased in the PC group. The results of Cao et al. (40) were consistent with the results of the present study, which may be related to the higher LBP concentration in *L. barbarum* leaves. The addition of different concentrations of the *L. barbarum* leaf homogenates increased the relative abundance of Bacteroidota. Ding et al. (28) fed cyclophosphamide-treated mouse LBP and observed an increase in the relative abundance of Bacteroidota in the mouse intestine, which was similar to the results obtained by Cui et al. (35). Similar results were found in the present study. Bacteroidota, the main bacterium involved in SCFA production, is an anaerobic gram-negative bacterium that improves host nutrient digestion and absorption by improving carbohydrate metabolism (41). The relative abundance of unidentified_Bacteria was rapidly reduced with low homogenate concentrations of *L. barbarum* leaves, which may be because unidentified bacteria inhibit the growth of active ingredients found in *L. barbarum* leaves, and the specific molecular mechanism of inhibition requires further study. The addition of high concentrations of *L. barbarum* leaf homogenate markedly increased the abundance of other bacteria, probably because of some specific active components in the high *L. barbarum* leaf homogenate group, which additionally favor bacterial growth.

*Akkermansia* and Romboutsia were the dominant genera, and the relative abundance of *Akkermansia* in the 2 g/kg LD group significantly increased. This may be related to the presence of LBP in *L. barbarum* leaves. Xu et al. (42) found an increase in the relative abundance of *Akkermansia* when rats were fed by gavage with leaves of different LBP, which is consistent with the results of the present study. LBP have been found to contribute to the colonization of beneficial microorganisms in the intestinal tract, improve intestinal barrier function, alleviate intestinal permeability, and maintain intestinal health (43). Beneficial bacteria in appropriate quantities contributes to the maintenance of the intestinal epithelial barrier and modulate immune homeostasis by competitively inhibiting pathogens and producing antimicrobial compounds, thereby reducing inflammation caused by harmful intestinal bacteria (44). *Akkermansia* regulates mucus thickness, intestinal barrier integrity, and immune function and is a beneficial bacterium that promotes health and has a positive effect on regulating host metabolic disorders (45). The abundance of Romboutsia and Candidatus_Saccharimonas was significantly lower in the MD group than in the PC group, which may be associated with the presence of LBP in the leaves. Liu et al. (46) reported that dietary supplementation of 400 mg/kg LBP significantly reduced the relative abundance of Romboutsia bacteria in the rat intestines, which was consistent with the results of the present study. Romboutsia ensures a healthy state in the intestine and is abundant in the healthy intestinal mucosa (47, 48). However, rats with acute necrotizing pancreatitis exhibit reduced relative abundance of Candidatus_Saccharimonas, suggesting that it plays a crucial role in maintaining normal intestinal function (49). In the present study, the relative abundance of Candidatus_Saccharimonas rapidly decreased possibly because some active components in *L. barbarum* leaves inhibited the growth of Candidatus_Saccharimonas. Specific functional substances need to be further studied. The relative abundance of Alloprevotella was significantly higher in the HD group than in the PC group, which may be related to the carbohydrate content of *L. barbarum* leaves. Wu et al. (50) showed that the abundance of Alloprevotella correlate with the addition of high-carbohydrate diets, which could be used to improve digestion in animals. The relative abundance of Oscillibacter was significantly higher in the HD group than in the other groups. Oscillibacter is involved in preventing inflammation by breaking down polysaccharides into butyrate. Furthermore, it exerts additional protective effects on the host by modulating abundant acid-producing bacteria to influence SCFA content in the rat intestine (51). Zhou et al. (52) reported that adding LBP to the diet significantly affected bacterial growth and secondary metabolite synthesis by influencing the structure of intestinal microflora. A link between the presence of certain beneficial bacteria in the animal guts and growth performance has previously been elucidated (53). Correlation analysis showed that the final weight and daily weight gain of the rats were significantly negatively correlated with Anaerostipes, Alloprevotella, and Phascolarctobacterium. The F/G ratio and daily food intake of the rats showed a significant positive correlation with Phascolarctobacterium and Alloprevotella. Phascolarctobacterium has been reported to produce SCFA, which is critical in regulating metabolic balance in the intestine. This genus is commonly found in the intestine and is beneficial to organisms (54). Zhang et al. (55) reported that the gram-negative bacteria Anaerostipes, Phascolarctobacterium, and Alloprevotella can aggravate intestinal inflammation, destroy intestinal structure, affect nutrient absorption, and ultimately reduce growth performance. Furthermore, butyrate has been reported to store 10–15% of the energy required by the host (56). A high butyrate concentration can have the opposite effect and damage the integrity of the intestinal barrier (57). In the present study, the growth performance of rats was significantly negatively correlated with the presence of Alloprevotella, Anaerostipes, and Phascolarctobacterium. In addition, daily food intake of rats was significantly positively correlated with the presence of some unclassified bacteria among other bacteria, that can promote nutrient digestion and absorption and thus improve the growth performance of rats.

## 5 Conclusion

Dietary supplementation with *L. barbarum* leaves in the diet improved the structure of intestinal flora, increased the relative abundance of beneficial bacteria in the gut microbiota, and enhanced the growth performance and organ weight in rats. The recommended dietary supplement level of *L. barbarum* leaves is 2 g/kg. However, rats were used as an experimental model in this study, and their application in animal production requires further studies especially in animals such as poultry and swine. In addition, the functional mechanism of *L. barbarum* leaves needs more investigation to provide a theoretical basis for its future application.

## Data availability statement

The original contributions presented in the study are publicly available. This data can be found here: China National Center for Bioinformation (CNCB), PRJCA028224.

## Ethics statement

The animal study was approved by Attitude of Ningxia University Technology Ethics Committee. The study was conducted in accordance with the local legislation and institutional requirements.

## Author contributions

YG: Conceptualization, Data curation, Formal analysis, Investigation, Methodology, Writing – original draft, Writing – review & editing. JL: Conceptualization, Formal analysis, Supervision, Writing – review & editing. QT: Conceptualization, Formal analysis, Supervision, Writing – review & editing. DZ: Conceptualization, Supervision, Writing – review & editing. MW: Supervision, Writing – review & editing. GX: Conceptualization, Funding acquisition, Supervision, Writing – review & editing.
